# Unilateral acute anterior uveitis is associated with ipsilateral changes in the tear fluid proteome that involves the LXR/RXR pathway

**DOI:** 10.1186/s12348-020-00204-4

**Published:** 2020-05-27

**Authors:** Jon Roger Eidet, Øystein Kalsnes Jørstad, Ida G. Fostad, Ole K. Olstad, Ragnhild Ø. Sørland, Morten C. Moe, Goran Petrovski, Milaim Pepaj

**Affiliations:** 1 Center for Eye Research, Department of Ophthalmology, Oslo University Hospital, University of Oslo, Oslo, Norway; 2 Faculty of Dentistry, Department of Oral Biology, University of Oslo, Oslo, Norway; 3 Blood Cell Research Group, Section for Research, Department of Medical Biochemistry, Oslo University Hospital, Oslo, Norway; 4 Hormone Laboratory, Department of Medical Biochemistry, Oslo University Hospital, Oslo, Norway

**Keywords:** Uveitis, Tear fluid, Proteomics

## Abstract

**Purpose:**

To determine whether unilateral acute anterior uveitis (AAU) induces ipsilateral changes in the tear fluid proteome.

**Methods:**

Five patients (25–77 years old) with unilateral AAU were included. Tear fluid samples were obtained using Schirmer’s test strips. The healthy eye served as control. Proteins were identified by liquid chromatography tandem mass spectrometry.

**Results:**

Two hundred forty-two tear fluid sample proteins were identified, of which 75 were present in at least three patients. Nine proteins were at least 1.5-fold increased, whereas eight were at least 1.5-fold decreased in tears from the diseased eye compared with the healthy eye. APOBEC3A was significantly increased (1.43-fold; *P* = 0.04), whereas TGM2 was significantly decreased (− 1.21-fold; *P* = 0.03) in tears from the diseased eye relative to the healthy eye. Ingenuity Pathway Analysis identified LXR/RXR (*P* < 1.02E−4) as a top canonical pathway.

**Conclusion:**

Unilateral AAU induced detectable changes in the ipsilateral tear fluid proteome and involvement of the inflammation-associated LXR/RXR pathway.

## Introduction

Uveitis denotes a heterogeneous group of diseases characterized by intraocular inflammation. Among the uveitis subtypes, inflammation most commonly involves the eye’s anterior segment (30–60% of all uveitis cases) [1]. Acute anterior uveitis (AAU) is usually unilateral in its presentation. AAU typically resolves following prompt and adequate ophthalmic care. A delayed diagnosis, however, is associated with an increased risk of potentially severe complications [2, 3]. The diagnosis of AAU typically requires ophthalmic work-up and is aided by slit-lamp examination. On the other hand, non-ophthalmologist doctors may easily misinterpret AAU as harmless external eye disease, e.g., common infectious conjunctivitis. As a consequence of a false diagnosis, adequate treatment may be delayed. Nevertheless, patients are often primarily admitted to general practitioners lacking access to slit-lamps and other ophthalmic instruments. Consequently, there is a need for a simple diagnostic method to diagnose AAU, thereby preventing delays in diagnosis, treatment, and subsequent complications.

Blood tests to detect inflammation are diagnostic cornerstones in a vast number of medical conditions. In AAU, however, blood analyses lack necessary sensitivity and specificity. On the other hand, diagnostic samples from the eye’s aqueous humor or vitreous body necessitate invasive techniques that pose a risk of serious ocular complications. By contrast, tear fluid sampling is non-invasive, harmless, and easily performed with minimal training. Moreover, elevated cytokine levels have been demonstrated in tear fluid of AAU patients [3]. Accordingly, there is a rationale for investigating the diagnostic potential of tear fluid biomarkers in AAU.

By virtue of advancing proteomic analysis methods, quantification of more than 1.500 tear fluid proteins can potentially be achieved [4]. Furthermore, in unilateral AAU, tear fluid samples from the healthy eye can serve as control and allow for validation of AAU-associated proteins. The basis for examining tear fluid proteins has been laid in studies of disorders like rosacea, dry eye syndromes, and Graves’ disease [5]. The tear fluid proteome in AAU is less explored, but some data do exist. First, one study assessed tear fluid cytokines and chemokines in uveitis patients [3]. It did not examine the full tear fluid proteome but instead used a multiplex bead analysis that assayed 21 predefined molecules. The authors concluded there were significant differences in the levels of several cytokine in uveitis patients compared with healthy controls. Secondly, in a conference abstract, Angeles-Han et al. reported results from a pilot study on proteome analysis of tear fluid obtained with Schirmer’s strips from children with idiopathic or juvenile idiopathic arthritis (JIA)-associated anterior uveitis [6]. The investigation detected 1.224 unique protein groups, of which 120 were significantly different from healthy controls. Finally, in a study on tear proteome by Chunky et al., lactoferrin and lipocalin were identified as promising biomarkers for JIA-associated uveitis [7]. Nevertheless, there are no well-defined tear fluid biomarkers for AAU, let alone a consensus about their diagnostic value.

To assess the potential use of tear fluid for diagnostic purposes in AAU, the current pilot study aimed at exploring whether unilateral AAU is associated with ipsilateral changes in the tear fluid proteome.

## Materials and methods

### Patients

The study included five adult patients diagnosed with unilateral AAU at the Department of Ophthalmology at Oslo University Hospital, Norway. All patients were examined by the same ophthalmologist, and the AAU diagnosis was based on (1) Findings: unilateral anterior chamber cells (obligate) and ipsilateral anterior chamber flare, ciliary injection, and miosis. (2) Symptoms: ocular pain and photophobia. Exclusion criteria were age < 18 years, pregnancy, bilateral AAU, intermediate-, posterior- or pan-uveitis, AAU secondary to suspected or known infectious disease, presence of any concurrent ocular disease (including dry eye syndrome, conjunctivitis, keratitis, eyelid disease, glaucoma, or neoplastic disease), prior severe ocular trauma, prior ocular surgery, intraocular pressure < 6 or > 21 mmHg, use of topical eye medication, use of systemic anti-inflammatory medication, or smoking.

### Tear fluid sampling

The research group used a modified version of a previously described protocol for utilizing Schirmer’s strips to examine tear fluid proteins [4, 5]. In brief, one Schirmer’s test strip was placed in each eye’s inferior fornix without concurrent anesthesia and left for 5 min with eyes closed. The strips were then immediately transferred to microcentrifuge tubes and stored at − 140 °C until analyses. To detect changes in the tear fluid proteome of the AAU eye, the healthy eye served as control.

### Protein extraction

Protein extraction was performed according to a protocol previously reported by the research group [4, 5].

### Ingenuity Pathway Analysis

To explore biological function, lists of protein identifiers (UniProt) were uploaded onto the web-delivered application Ingenuity Pathways Analysis (IPA) (QIAGEN N.V., Venlo, Netherlands).

### Statistical analysis

The R statistical software (R Development Core Team, R 3.4.2 GUI 1.70 El Capitan build [7434]) was used for statistical analyses; a one-sample *t* test was used to compare the AAU eyes with the respective healthy controls.

## Results

### Patients

The patients’ clinical data are presented in Table 1.

**Table 1:** Patients with unilateral acute anterior uveitis included in the study

	Patient 1	Patient 2	Patient 3	Patient 4	Patient 5
Age (years)	28	57	41	25	77
Sex	Male	Male	Female	Male	Female
Ethnicity	Caucasian	Caucasian	Caucasian	Caucasian	Caucasian
HLA-B27 status	Unknown	HLA-B27 negative	HLA-B27 positive and palmoplantar psoriasis	Unknown	HLA-B27 positive
Previous episodes of AAU	No	No	No	Yes	No
Duration of symptoms prior to tear fluid sampling (days)	1	10	5	1	7
AC flare (laser flare photometry)	47/4	3/54	129/3	11/3	*
AC flare (Sun criteria)	§	0/+ 2	+ 2/0	+ 1/0	+ 4/0
AC cells (Sun criteria)	§	0/+ 1	+ 1/0	± /0	+ 4/0
Corneal precipitates	§	Non-granular	Non-granular	None	Non-granular
AC fibrin	No	No	No	No	Yes
AC hypopyon	No	No	No	No	No
Posterior synechiae	No	No	No	No	Yes
Cells in anterior vitreous	No	No	No	No	#
Decimal visual acuity	1.0/1.0	1.0/1.0−−	1.2++/1.5−−	1.2++/1.2++	CF/1.0−−
Intraocular pressure (mmHg)	14/17	13/7	10/14	7/10	20/10
Schirmer test type 1 (0–40; mm)	40/40	33/40	17/7	7/7	30/10

Bilateral measurements are presented as right eye/left eye

*AAU* acute anterior uveitis, *AC* anterior chamber, *CF* counting fingers

*Measurement not obtained

§The standardization of uveitis nomenclature criteria not reported

# Posterior segment not visible

### Relative tear fluid protein levels in the diseased eye versus the healthy eye

Relative protein ratios were calculated for 242 tear fluid proteins present in both the diseased and the healthy eye. Of these nine were at least 1.5-fold increased, and eight were at least 1.5-fold decreased in the diseased eye compared with the healthy control. Apolipoprotein B mRNA editing enzyme catalytic subunit 3A (APOBEC3A) was significantly increased in the diseased eye compared with the healthy control (fold change 1.43; *P* = 0.04), whereas transglutaminase 2 (TGM2) was significantly decreased in the diseased eye compared with the healthy control (fold change − 1.21; *P* = 0.03). Table 2 summarizes the relative ratios of the top 20 upregulated proteins for each of the five patients.

**Table 2:** Top 20 upregulated protein level ratios found in tear fluid of the diseased eye relative to the healthy eye of patients with unilateral acute anterior uveitis

Patient 1		Patient 2		Patient 3		Patient 4		Patient 5	
Protein symbol	*RR*	Protein symbol	*RR*	Protein symbol	*RR*	Protein symbol	*RR*	Protein symbol	*RR*
SERPINA3	14.73	ZG16B	5.62	DGKZ	9.13	KRT32	8.96	FGB	5.33
CEP104	4.32	GAPDH	3.17	SCGB2A1	6.19	PTCHD1	2.70	APOH	4.87
RARRES1	3.29	LTF	2.89	PLA2G2A	6.04	EEF1A1	2.55	OPRPN	3.85
ALDH3A1	2.85	DCD	2.86	SCGB1D1	4.19	TPI1	2.33	A2M	3.79
ADIRF	2.68	OPRPN	2.70	WWC3	3.64	ADIRF	1.96	C4A/C4B	2.57
KRT10	2.59	S100P	2.46	LYZ	3.58	S100A11	1.95	WWC3	2.49
CST1	2.46	GSN	2.12	OPRPN	3.35	GSTP1	1.91	HPR	2.33
FABP5	2.20	SCGB1D1	1.98	PSME2	2.65	SCGB1D1	1.88	C3	2.17
ADH7	2.19	EEF1A1	1.88	CST1	2.54	ANXA2	1.83	APOA1	2.05
CP	2.06	AZGP1	1.82	LACRT	2.35	LDHA	1.68	ORM2	2.00
TMSB10/TMSB4X	1.76	TXN	1.80	MASP1	2.27	IL1RN	1.64	A1BG	1.81
IL1RN	1.75	PIP	1.79	B2M	2.03	GPI	1.62	HPX	1.62
RPLP1	1.70	WWC3	1.69	A1BG	1.91	PSME2	1.62	TF	1.50
SFN	1.70	LCN1	1.52	LCN1	1.76	IGHG2	1.60	PIP	1.48
SH3BGRL	1.63	LYZ	1.43	LTF	1.75	APOBEC3A	1.59	CLU	1.45
WFDC2	1.57	ALB	1.22	S100A6	1.73	B2M	1.58	DMBT1	1.44
CTIF	1.55	C3	1.16	HPX	1.71	FGG	1.58	CST1	1.40
DBI	1.53	PKM	1.14	CST4	1.67	CFL1	1.53	SERPINA3	1.34
YWHAE	1.50	ACTB	1.13	LGALS3	1.67	SERPINB1	1.50	IGHG2	1.33
ACTN4	1.49	CLU	1.12	PIP	1.66	ATG9A	1.50	PFN1	1.25

*RR* relative ratio of protein level in tear fluid of diseased versus healthy eye

**Fig. 1:**
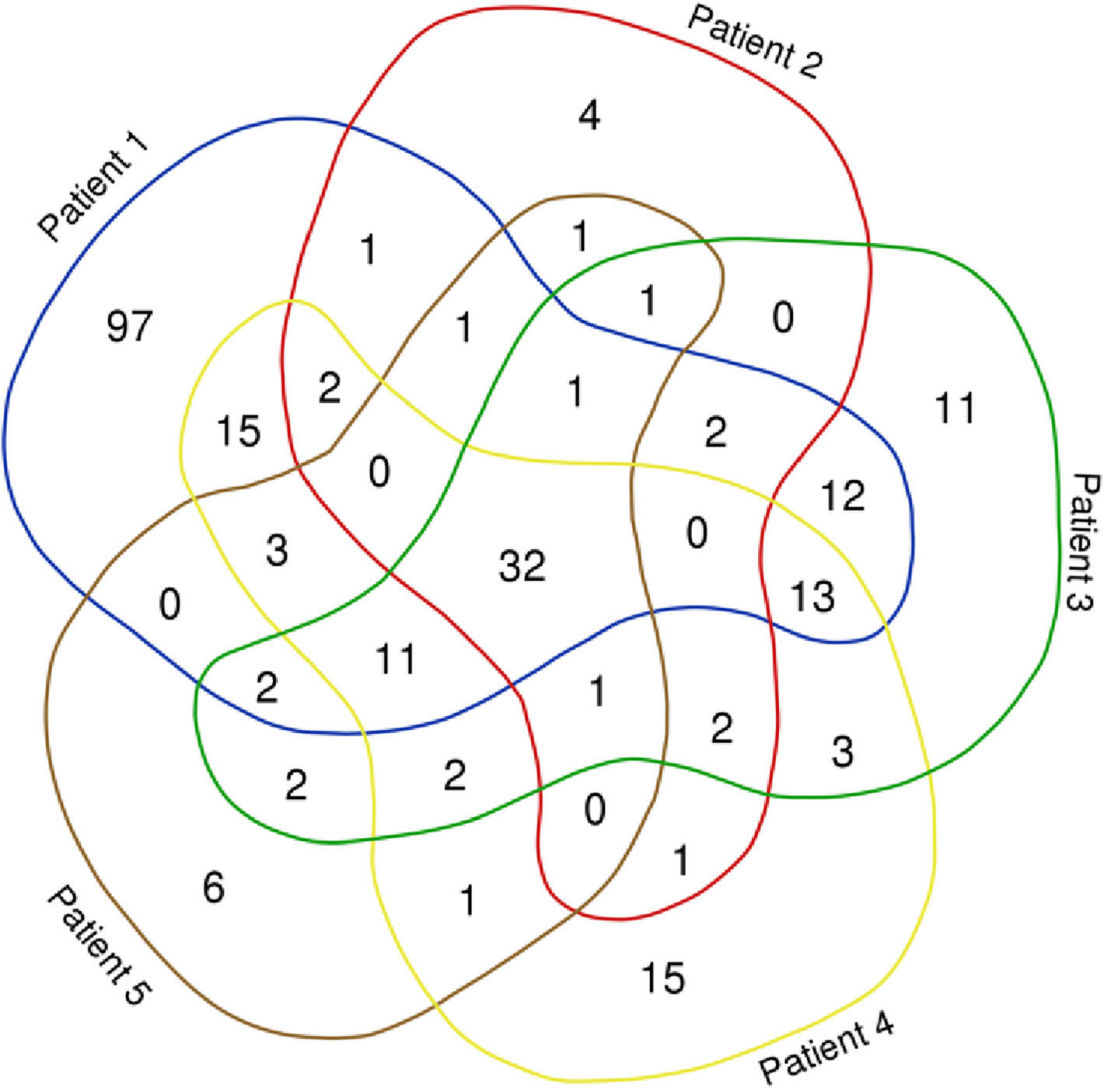
Venn diagram illustrating the number and distribution of matching tear fluid proteins in five patients with acute anterior uveitis [8]

Thirty-two tear fluid proteins were identified in all five patients; the number of matching proteins was highest between patients 1, 3, and 4 (Fig. 1).

### Ingenuity Pathway Analysis

For all patients, IPA identified the liver X receptor/retinoid X receptor (LXR/RXR) and the farnesoid X receptor/retinoid X receptor (FXR/RXR) among the “Top Canonical Pathways” affected in the tear fluid (Table 3).

**Table 3:** Top canonical pathways

Patient 1		Patient 2		Patient 3		Patient 4		Patient 5	
Protein	*P* value	Protein	*P* value	Protein	*P* value	Protein	*P* value	Protein	*P* value
LXR/RXR activation	2.04E−12	Glycolysis I	2.06E−07	LXR/RXR activation	3.14E−15	LXR/RXR activation	3.11E−18	LXR/RXR activation	2.92E−19
Glycolysis I	3.13E−11	LXR/RXR activation	1.02E−04	FXR/RXR activation	1.69E−13	Acute phase response signaling	5.67E−16	FXR/RXR activation	2.88E−17
FXR/RXR activation	8.01E−10	Clathrin-mediated endocytosis signaling	6.79E−04	Acute phase response signaling	2.32E−09	FXR/RXR activation	7.25E−15	Acute phase response signaling	1.53E−15
Gluconeogenesis I	1.61E−09	Gluconeogenesis I	1.25E−03	Atherosclerosis signaling	2.97E−09	Glycolysis I	1.24E−11	Clathrin-mediated endocytosis signaling	5.04E−09
Acute phase response signaling	2.36E−09	FXR/RXR activation	2.08E−03	Clathrin-mediated endocytosis signaling	1.06E−08	Atherosclerosis signaling	3.63E−09	Glycolysis I	1.01E−08

Upstream regulator analysis in IPA aims to identify transcriptional regulators that can explain the observed changes in the dataset. IPA identified interleukin 6 (IL6) and/or tumor necrosis factor (TNF) as possible upstream regulators in four of the five patients (Table 4).

**Table 4:** Top upstream regulators

Patient 1		Patient 2		Patient 3		Patient 4		Patient 5	
Protein	*P* value	Protein	*P* value	Protein	*P* value	Protein	*P* value	Protein	*P* value
MAPT	1.32E−24	APP	5.03E−11	MAPT	7.78E−15	IL6	1.65E−16	MAPT	5.83E−12
APP	1.78E−22	MAPT	5.49E−11	APP	6.79E−13	APP	1.46E−14	APP	1.72E−11
TP53	3.21E−19	PSEN1	2.71E−09	TNF	8.03E−13	MAPT	2.12E−13	ZNF106	3.96E−11
TNF	9.00E−19	Growth hormone	2.87E−07	PSEN1	3.75E−12	PCGEM1	4.90E−12	PSEN1	6.04E−10
PSEN1	1.40E−17	MYC	7.50E−07	IL6	1.04E−11	TNF	5.37E−11	IL6	9.72E−10

Furthermore, for all patients IPA recognized “inflammatory response” and “immune cell trafficking” among the top categories within “Diseases and Bio functions” (Tables 5 and 6).

**Table 5:** Top diseases and bio functions—diseases and disorders

Patient 1		Patient 2		Patient 3		Patient 4		Patient 5	
Protein	*P* value	Protein	*P* value	Protein	*P* value	Protein	*P* value	Protein	*P* value
Dermatological diseases and conditions	1.25E−04 to 3.17E−25	Inflammatory response	4.02E−03 to 1.91E−10	Inflammatory response	3.49E−04 to 9.03E−20	Metabolic disease	2.52E−04 to 1.54E−16	Metabolic disease	2.69E−03 to 1.27E−14
Organismal injury and abnormalities	2.03E−04 to 3.17E−25	Organismal injury and abnormalities	4.02E−03 to 1.91E−10	Organismal injury and abnormalities	3.49E−04 to 9.03E−20	Connective tissue disorders	3.72E−04 to 6.67E−16	Inflammatory response	2.55E−03 to 2.94E−14
Metabolic disease	1.89E−04 to 3.65E−23	Hematological disease	3.44E−03 to 3.53E−10	Metabolic disease	3.18E−04 to 7.41E−18	Inflammatory disease	5.16E−04 to 6.67E−16	Hematological disease	2.32E−03 to 2.21E−13
Inflammatory response	1.87E−04 to 1.24E−21	Immunological disease	3.44E−03 to 3.53E−10	Neurological disease	2.91E−04 to 6.16E−15	Inflammatory response	5.16E−04 to 6.67E−16	Immunological disease	1.95E−03 to 2.21E−13
Neurological disease	2.03E−04 to 1.37E−21	Inflammatory disease	2.94E−03 to 3.53E−10	Psychological disorders	2.55E−04 to 6.16E−15	Organismal injury and abnormalities	5.16E−04 to 6.67E−16	Inflammatory disease	2.55E−03 to 2.21E−13

**Table 6:** Top diseases and bio functions—physiological system development and function

Patient 1		Patient 2		Patient 3		Patient 4		Patient 5	
Protein	*P* value	Protein	*P* value	Protein	*P* value	Protein	*P* value	Protein	*P* value
Immune cell trafficking	1.23E−04 to 3.97E−15	Immune cell trafficking	4.02E−03 to 1.33E−08	Immune cell trafficking	3.34E−04 to 2.69E−16	Immune cell trafficking	5.24E−04 to 2.61E−13	Immune cell trafficking	2.48E−03 to 2.24E−11
Hematological system development and function	2.01E−04 to 1.33E−13	Hematological system development and function	4.02E−03 to 5.37E−07	Hematological system development and function	3.34E−04 to 1.79E−12	Hematological system development and function	5.24E−04 to 4.87E−12	Hematological system development and function	2.55E−03 to 1.31E−09
Cardiovascular system development and function	1.66E−04 to 1.20E−11	Humoral immune response	2.42E−03 to 2.83E−06	Tissue morphology	3.21E−04 to 5.10E−10	Cardiovascular system development and function	5.24E−04 to 1.56E−10	Organismal development	1.88E−03 to 2.65E−07
Organismal development	1.66E−04 to 5.69E−11	Organismal development	4.02E−03 to 3.65E−05	Organismal development	2.62E−04 to 5.04E−09	Organismal development	4.95E−04 to 2.22E−10	Humoral immune response	9.19E−04 to 2.78E−07
Humoral immune response	6.88E−05 to 2.84E−10	Lymphoid tissue structure and development	3.90E−03 to 4.99E−05	Organismal survival	3.81E−06 to 8.49E−09	Organismal survival	1.89E−05 to 1.27E−08	Tissue morphology	2.27E−03 to 4.15E-07

## Discussion

The current pilot study identifies ipsilateral changes in the tear fluid proteome of patients with unilateral AAU. Accordingly, Schirmer’s test sampling of tear fluid potentially represents an adjuvant non-invasive approach to investigate the pathophysiological pathways in AAU.

For all patients, IPA identified the LXR/RXR and FXR/RXR pathways among the top canonical pathways affected. LXRs have predominantly been linked to regulation of lipid metabolism, but there is also mounting evidence of an anti-inflammatory role [9]. LXRs modulate the immune response in lipopolysaccharide-activated macrophages by inhibiting expression of inflammatory mediators, including IL6 [10]. LXR activation has also been associated with inhibition of the nuclear transcription factor-κB (NF-κB) [11], which is a major transcription factor in the regulation of immune responses. In rats, inhibition of NF-κB ameliorated anterior uveitis in experimental autoimmune uveitis (EAU) [12]. Furthermore, in a mouse model of EAU, a synthetic LXR agonist reduced ocular inflammation [13]. The authors of the latter study suggested that LXR agonists had potential for becoming a therapeutic option for uveitis patients. LXRs were expressed in the native rat corneal epithelium and stroma in a rat model of inflammatory corneal angiogenesis [14]. Following induction of inflammatory corneal angiogenesis, LXR expression temporarily decreased in the corneal epithelium, while removal of the inflammatory stimulus (corneal suture) was associated with activation of the LXR/RXR pathway and resolution of inflammation. Consequently, the authors suggested that LXR/RXR pathway activation could contribute to resolution of corneal inflammation. To our knowledge, the FXR/RXR pathway has not been directly linked to the pathogenesis of uveitis. In a study on endotoxin-induced uveitis in rats, however, the FXR antagonist guggulsterone suppressed ocular inflammation [15]. The authors related this effect to the inhibition of NF-κB by guggulsterone. Ultimately, engagement of the LXR/RXR and FXR/RXR pathways in the current study indicates that signs of AAU-associated inflammation are detectable in ipsilateral tear fluid samples.

In the current study, IPA determined that the changes to the tear proteome in AAU were associated with an inflammatory process (i.e., *inflammatory response* and *immune cell trafficking* were among the *top diseases and bio functions*). The upstream analysis by IPA further underscored the inflammatory tear profile by identifying TNF and IL6 among the top upstream regulators in four of the five patients. The role of TNF in the pathogenesis of uveitis has been well-established in animal studies [16, 17], and the concentration of TNF was also raised in the aqueous humor of patients with uveitis [18]. Most importantly, the essential role of TNF in uveitis has been confirmed by the recent success of employing the TNF-inhibitor adalimumab in the treatment of non-infectious uveitis [19, 20]. IL6 promotes the differentiation of CD4+ T-helper (Th) cells into Th17 cells, thereby contributing to the pathogenesis of autoimmune diseases [21]. In uveitis, IL6 has been proposed to be especially important in HLA-B27 positive cases [22]. The latter study also reported a higher concentration of IL6 in the aqueous humor of HLA-B27 positive uveitis patients compared to patients with Vogt-Koyanagi-Harada disease, sarcoidosis, or idiopathic granulomatous uveitis. This is in line with our study, in which IPA identified IL6 as an upstream regulator in the two HLA-B27 positive patients.

We found a significantly higher level of APOBEC3A, a DNA cytidine deaminase, in tear fluid of the diseased eye relative to the healthy control. Interferon, a regulating cytokine in autoimmune uveitis [23], can induce APOBEC3A in monocytes. This enables deamination of foreign double-stranded DNA and inhibits replication of retroviruses and retrotransposons [24]. In this way, APOBEC3A partakes in the innate immune system, and its increase in tear fluid from AAU eyes indicates local disease activity.

The level of TGM2 was reduced in tear fluid from the diseased eye. This is contradictory to a study on endotoxin-induced uveitis, in which the level of TGM2 was increased in aqueous humor [25]. In agreement with our results, however, studies on Crohn’s disease patients have reported an inverse relationship between TGM2 and disease activity [26].

In total, 242 unique proteins were identified in the tear fluid samples. Only 32 proteins, however, were identified in all patients. The highest number of matching tear fluid proteins was seen in the three youngest patients that also had the shortest disease duration. On the other hand, HLA-B27 status was not obviously related to the number of matching tear fluid proteins. Nevertheless, as the anterior chamber cytokine profile differs between HLA-B27 positive and negative patients, the heterogeneity in HLA-B27 status might have contributed to the relative lack of matching proteins between patients in the current study.

In our study, tear fluid proteins were identified using a single Schirmer’s test strip in each patient. Previous studies have reported a higher number of identified proteins when pooling tear fluid samples from multiple patients [5]. Although proteomics is useful as a screening method for AAU biomarkers, the method has limited sensitivity for low-concentration proteins. Alternative methods, including enzyme-linked immunosorbent assay, are more suitable for detecting proteins at low concentrations. Accordingly, restricted sensitivity may have contributed to the rather poor correspondence of tear fluid proteins between the patients in the current study. Conclusively, we believe that failure to identify certain proteins across all patients in the present study should not lead to the exclusion of these as potential biomarkers.

Serpent family A member 3 (SERPINA3), also known as serine protease inhibitor 3 (SPI-3), was more than 14-fold elevated in tear fluid from the diseased eye in one patient, despite disease duration of only a single day. In addition, this protein was mildly elevated in two other patients. SERPINA3, also referred to as SPI-3, is normally found in the aqueous humor [27], but it is also part of the acute phase response. In endotoxin-induced uveitis in rats following lipopolysaccharide injection, SERPINA3 mRNA in epithelial cells of the iris and ciliary body was rapidly upregulated as part of the initial inflammatory response [28]. Yet another potential biomarker protein was increased in tear fluid from the diseased eye in three patients: orosomucoid 2 (ORM2), also known as alpha-1-acid glycoprotein. Similar to SERPINA3, ORM2 is an acute phase response protein. In a previous study, serum levels of ORM2 were increased in moderate and severe idiopathic AAU [29]. Furthermore, in the latter study, ORM2 levels correlated with disease severity and returned to normal following resolution.

In conclusion, unilateral AAU induces inflammation-associated changes in the ipsilateral tear fluid proteome; in a clinical setting, Schirmer’s strips can be used to collect tear samples for proteomics. The inflammation-associated LXR/RXR was among the top canonical pathways affected. Further studies are necessary to determine whether APOBEC3A, SERPINA3, and ORM2 are potential AAU biomarkers.

## Data Availability

Not applicable.

## Abbreviations

AAU: Acute anterior uveitis
AC: Anterior chamber
APOBEC3A: Apolipoprotein B mRNA editing enzyme catalytic subunit 3A
CF: Counting fingers
EAU: Experimental autoimmune uveitis
FXR/RXR: Farnesoid X receptor/retinoid X receptor
IPA: Ingenuity Pathways Analysis
JIA: Juvenile idiopathic arthritis
LXR/RXR: Liver X receptor/retinoid X receptor
NF-κB: Nuclear transcription factor-κB
ORM2: Orosomucoid 2
SERPINA3: Serpent family A member 3
SPI-3: Serine protease inhibitor 3
TGM2: Transglutaminase 2
